# Towards improving the identification of anterior cruciate ligament tears in primary point-of-care settings

**DOI:** 10.1186/s12891-020-03237-x

**Published:** 2020-04-17

**Authors:** Jackie L. Whittaker, Michelle Chan, Bo Pan, Imran Hassan, Terry Defreitas, Catherine Hui, Luciana Macedo, David Otto

**Affiliations:** 1 Department of Physical Therapy, Faculty of Medicine, University of British Columbia, 2177 Westbrook Mall, Vancouver, V6T 1Z3 Canada; 2 Arthritis Research Canada, Richmond, Canada; 3 Department of Physical Therapy, Faculty of Rehabilitation Medicine, University of Alberta, Edmonton, Canada; 4 Glen Sather Sports Medicine Clinic, University of Alberta, Edmonton, Canada; 5 EPICORE Centre & Alberta SPOR Support Unit, Consultation & Research Services, University of Alberta, Edmonton, Canada; 6 Department of Family Medicine, Faculty of Medicine and Dentistry, University of Alberta, Edmonton, Canada; 7 Department of Surgery, Faculty of Medicine and Dentistry, University of Alberta, Edmonton, Canada; 8 School of Rehabilitation Science, McMaster University, Hamilton, Canada

**Keywords:** Diagnosis, Family medicine, Knee trauma, Physical therapy

## Abstract

**Background:**

Only a small proportion of anterior cruciate ligament (ACL) tears are diagnosed on initial healthcare consultation. Current clinical guidelines do not acknowledge that primary point-of-care practitioners rely more heavily on a clinical history than special clinical tests for diagnosis of an ACL tear. This research will assess the accuracy of combinations of patient-reported variables alone, and in combination with clinician-generated variables to identify an ACL tear as a preliminary step to designing a primary point-of-care clinical decision support tool**.**

**Methods:**

Electronic medical records (EMRs) of individuals aged 15–45 years, with ICD-9 codes corresponding to a knee condition, and confirmed (ACL^+^) or denied (ACL^−^) first-time ACL tear seen at a University-based Clinic between 2014 and 2016 were eligible for inclusion. Demographics, relevant diagnostic indicators and ACL status based on orthopaedic surgeon assessment and/or MRI reports were manually extracted. Descriptive statistics calculated for all variables by ACL status. Univariate between group comparisons, clinician surveys (*n* = 17), availability of data and univariable logistic regression (95%CI) were used to select variables for inclusion into multivariable logistic regression models that assessed the odds (95%CI) of an ACL-tear based on patient-reported variables alone (consistent with primary point-of-care practice), or in combination with clinician-generated variables. Model performance was assessed by accuracy, sensitivity, specificity, positive and negative predictive values, and positive and negative likelihood ratios (95%CI).

**Results:**

Of 1512 potentially relevant EMRs, 725 were included. Participant median age was 26 years (range 15–45), 48% were female and 60% had an ACL tear. A combination of patient-reported (age, sport-related injury, immediate swelling, family history of ACL tear) and clinician-generated (Lachman test result) variables were superior for ACL tear diagnosis [accuracy; 0.95 (90,98), sensitivity; 0.97 (0.88,0.98), specificity; 0.95 (0.82,0.99)] compared to the patient-reported variables alone [accuracy; 84% (77,89), sensitivity; 0.60 (0.44,0.74), specificity; 0.95 (0.89,0.98)].

**Conclusions:**

A high proportion of individuals without an ACL tear can be accurately identified by considering patient-reported age, injury setting, immediate swelling and family history of ACL tear. These findings directly inform the development of a clinical decision support tool to facilitate timely and accurate ACL tear diagnosis in primary care settings.

## Background

Anterior cruciate ligament (ACL) tears are common in persons between the ages of 15–30 years, with an overall incidence estimated at 30 to 80 injuries per 100,000 persons (general population) [1–4]. These injuries most commonly occur during sport and recreational activities that involve frequent cutting, pivoting and jumping [3, 5, 6]. ACL tears are associated with reduced function [7–9], physical inactivity, increased risk of further injury [10], and future obesity [11] and/or osteoarthritis (OA) [12–14].

Although there is controversy regarding the need for, and timing of reconstructive surgery following an ACL tear [15], it is well established that an early and accurate diagnosis is vital to ensuring timely and appropriate treatment, which in turn improves both immediate (e.g., return to work, return to sport) and long-term (e.g., physical activity) outcomes [16]. Current clinical guidelines for full-thickness ACL tear diagnosis recommend obtaining a relevant history and performing a thorough clinical examination [17, 18]. Typically, a diagnosis is achieved by considering the mechanism of injury, immediacy of symptoms, pain location, observation, palpation, and outcome of special clinical tests [3, 19–21]. Experienced practitioners express confidence in diagnosing ACL tears without the need for diagnostic imaging which many consider to be a superfluous expense [22], and due to a lack of accessibility in some health systems, a preventable barrier to treatment [23].

Previous investigations have reported the diagnostic accuracy of a clinical examination for identifying a full-thickness ACL tear as good (i.e., sensitivity 0.77–0.99, specificity of 0.73–1.00) when performed by a healthcare practitioner with advanced orthopaedic training [21, 23]. Not surprisingly, the diagnostic accuracy of a clinical examination varies between orthopedic surgeons (94% accuracy) and primary care physicians (62% accuracy), with surgeons relying on both the clinical history and physical examination, and primary care physicians relying more heavily on the clinical history alone [24]. Differences in the weighting of clinical examination components for ACL tear diagnosis between practice settings likely reflect disparities in time between injury and examination, proportion of caseload comprised of acute knee injuries and confidence in performing clinical tests, amongst other factors.

Only 6.8 to 28.2% of ACL tears are diagnosed on initial healthcare consultation, with many patients waiting months for a correct diagnosis [3, 25–29]. Missed or falsely diagnosed ACL tears may result in delayed or misdirected rehabilitation, physician, and specialist visits and diagnostic imaging. At an individual level, misdiagnosis can lead to reduced mobility and knee confidence, physical inactivity, delayed return to activity (e.g., work or sport/recreation) and an increased risk of subsequent injuries, (e.g., meniscus tear) [3, 30, 31] and disease (e.g., post-traumatic OA) [32]. To facilitate timely and appropriate treatment, while minimizing individual patient and healthcare system burden, it is essential that primary point-of-care healthcare practitioners (e.g., emergency room physicians, family medicine physicians, physiotherapists) can accurately diagnose a full-thickness ACL tear early after injury.

Although the clinometric properties of individual diagnostic tests for full-thickness ACL tears [33, 34], and the diagnostic value of common clinical signs and symptoms [17] have been examined, previous investigations suffer from methodological limitations (i.e., small sample sizes), risk of selection bias [20, 23, 24, 28, 35–39] and have not acknowledged differences in the approach to ACL tear diagnosis between primary point-of-care practitioners and those with advanced orthopaedic training. Currently, there is no consensus about which combination of demographic characteristic(s), or clinical history and/or examination component(s) are the most valuable for the inclusion or exclusion of an ACL tear diagnosis that considers differences in practice patterns and competencies of primary point-of-care practitioners. A better understanding of the best combination of patient-reported variables, independent and in combination with clinician-generated variables for diagnosis of a full-thickness ACL tear could inform the development and evaluation of a primary point-of-care clinical decision tool aimed at facilitating early and accurate diagnosis. The primary objective of this study is to develop and internally validate two preliminary statistical models aimed at predicting full-thickness ACL tears based upon the best combination of patient-reported variables only or in combination with clinician-generated variables. We hypothesize that both statistical models will hold diagnostic value.

## Methods

### Study design and setting

This is a retrospective cohort study of the Electronic Medical Records (EMRs) of all patients, aged 15 to 45 years, who saw one of 11 primary care sport and exercise medicine primary care physicians or four orthopaedic surgeons at a multidisciplinary University-based community Sports Medicine Clinic for a knee condition between January 1, 2014 and June 30, 2016. Referrals to this clinic are from general practice physicians and physiotherapists. This study is reported as recommended by the Standards for Reporting Diagnostic Accuracy Studies 2015 (STARD) [40].

### Data source

Eligible individual EMRs were identified through a systematic search of the clinic’s EMR system (©HealthQuest). All records with a pre-determined International Classification of Disease-9 (ICD-9) code corresponding to a knee condition (see Additional file 1) with a confirmed or denied first-time full-thickness ACL tear were included. EMRs were excluded if the knee injury occurred prior to the pre-established study dates, represented a chronic ACL deficiency or re-tear of the ACL, or if the recorded diagnosis was knee OA. Prior to the study it was determined that a sample of 460 individual patient EMRs would provide appropriate statistical power (1-β = 0.8) based on 10 events per variable [41] for the outcome of interest (ACL tear), accounting for collinearity and assuming 55% EMR completion, 45% of completed EMRs represent individuals with ACL tears, and a predictive model (α = 0.05) of 10 independent variables.

### Reference standard

ACL tear diagnosis was based on a composite reference standard consisting of orthopaedic surgeon diagnosis and/or MRI findings to minimize misclassification bias. Diagnoses were dichotomized into full thickness ACL tear (ACL^+^; Lachman grade III) and no full-thickness ACL tear (ACL^−^; Lachman grade 0, I and II). Given that the sensitivity of clinical examination and MRI for identifying an ACL tear are 0.77–0.99 [21, 23] and 0.84–0.90 [42] respectively, there may be limitations to this criterion. However, as not all patients with an intra-articular knee injury require surgery, this combination was the most viable for identifying true negatives and false positives while minimizing verification bias.

### Procedures

Relevant data were manually extracted from the physician and/or surgeon chart notes, letters, diagnostic imaging and surgical reports contained within eligible EMRs. These data were compiled by research personnel and then audited for accuracy by one research team member (MC) using a custom designed data extraction tool (REDCap v6.17.2,©2017 Vanderbilt University). Extracted data included: 1) demographic characteristics (i.e., age, sex, height, weight, sport at time of injury if applicable), 2) injury details (i.e., date of injury, time since injury, time to diagnosis), 3) diagnostic details (i.e., ICD-9 code, surgeon diagnosis, diagnostic imaging study type, diagnostic imaging referring professional, diagnostic imaging findings), and 4) potential ACL tear diagnostic indicators (see Table 1).

**Table 1:** Summary of potential diagnostic indicators extracted from electronic medical records

Clinical History - Mechanism of Injury	contact or non-contact
	hyperextension, hyperflexion, plant/pivot, varus or valgus motion
Clinical History - Time of Injury	heard or felt a ‘pop’
	immediate (< 30 min) or delayed pain
	immediate (< 4 h) or delayed swelling
	inability to return to current activity
	knee catching, locking or instability
	inability to weight bear
Clinical Knee Examination	flexion and extension range of motion
	Lachman, Anterior Drawer and Pivot Shift test result
	valgus and varus stress test result
	Posterior Drawer test result
	McMurray, Apprehension test result
	presence and location of joint line pain
	presence and location of pain with palpation

Potential diagnostic indicators (i.e., mechanism of injury, clinical history and examination elements) were identified through a search of relevant literature and an anonymous healthcare practitioner survey (Additional file 2). Specifically, healthcare practitioners and consultants (i.e., physiotherapists, primary care sport and exercise medicine physicians and orthopaedic surgeons) were provided with an anonymous link to a short survey which asked them to identify and then rank what demographic characteristic(s), and/or clinical history and examination component(s) they felt were the most valuable for diagnosing a full-thickness ACL tear. Respondents ranked items within categories (i.e., patient characteristics, mechanism of injury, patient-reported symptoms at time of injury, clinical history responses, and clinical examination tests) in order of perceived importance. Potential diagnostic indicators that were ranked in the top tertile by at least one third of respondents were included in the data extraction tool.

### Statistical analysis

Analyses were performed using STATA© (v14.2, Collage Station, Texas, USA), R© 3.4.0. (Vienna, Austria) and SAS© 9.4 (Cary, NC, USA). Descriptive statistics including mean (95%CI), median (range), or proportion (exact 95%CI) were used to compare characteristics (i.e., age and sex) between patients represented by EMRs that did and did not meet the study inclusion criteria. For included EMR’s, missing data were identified and descriptive statistics including mean (95%CI), median (range) or proportion (exact 95%CI) were reported for all participant characteristics, potential diagnostic indicators and treatment pathway information variables by ACL status (study group) as appropriate. Univariable analyses (i.e., independent t-tests, Wilcoxon rank sum test, chi-square or Fisher’s test) were used to compare participant characteristics, potential diagnostic indicators and treatment pathway information by study group, as appropriate, accounting for multiple comparisons (*p* = 0.05/43 = 0.001). Assumptions of statistical tests were satisfied and the normality of continuous variable was tested with Normal Quantile-Quantile plots. The results of the healthcare practitioner survey were tabulated. Univariable logistic regression [odds ratio (OR), 95%CI] assessing the odds of an ACL tear were performed for selected variables that significantly differed between study groups on univariable analyses (i.e., *p*-value < 0.001) taking into consideration data availability and whether or not they were considered relevant based on the practitioner survey or literature review.

Multivariable logistic regression models were conducted with all prioritized variables and odds ratios (OR; 95%CI) for each variable in the final regression models were calculated. Items prioritized for inclusion into multivariable logistic regression models included the most promising patient-reported and clinician-generated predictors based on univariable logistic regression (*p* < 0.10). An ‘a priori’ decision was made to develop two multivariable logistic regression models for identifying an ACL tear. The first model included patient-reported predictors only to be consistent with primary point-of-care practice settings, while the second model included patient-reported and clinician-generated predictors. All assumptions for regression analyses were assessed. Multivariable models were built on a training set (random 70% of data) and evaluated with a test set (remaining 30% of data). To assess the performance of the models, accuracy rate (95%CI), the area under (AUC) the receiver-operating characteristic (ROC) curve, sensitivity (95%CI), specificity (95%CI), positive and negative predictive values (95%CI) and positive (LR+) and negative (LR-) likelihood ratios (95%CI) were calculated.

## Results

**Fig. 1:**
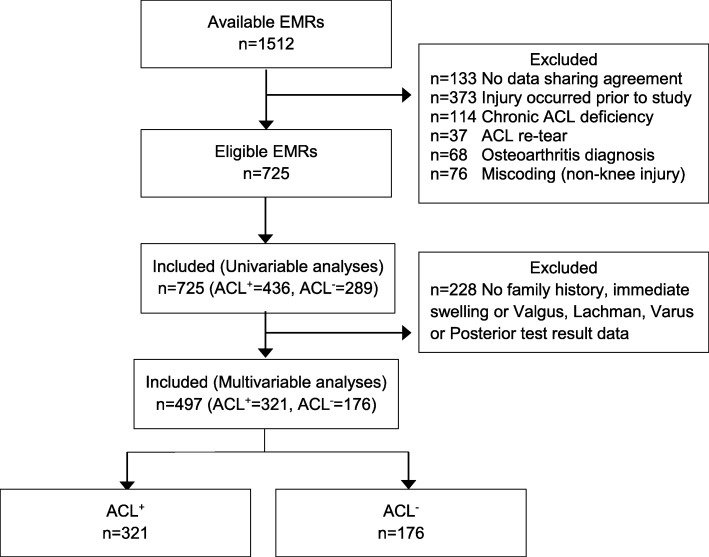
STARD diagram of the flow of Electronic Medical Records through the study

Of 1512 eligible individual EMRs, 725 met inclusion criteria (Fig. 1). Reasons for excluding EMRs included: no physician data sharing agreement (*n* = 133); injury occurred prior to January 2014 (*n* = 373); chronic ACL deficiency (*n* = 114); ACL re-tear (*n* = 37); osteoarthritis (*n* = 68); and mis-coding (i.e., a non-knee injury being coded as a knee injury) (*n* = 76). There was no difference in sex (included; 47.7% female, 95%CI 44.1, 51.4: excluded; 42.4% female, 95%CI 39.0, 45.9) or age (included; median age 26 years, range 15–45: excluded; median age 29 years, range 15–45) between the patients whose EMRs were included and those that were excluded.

The demographic characteristics and availability of clinical criterion standard data of all participants’ records that met inclusion criteria (*n* = 725) are presented in Table 2. The majority of participants had undergone an MRI (89.8%), 68.1% had been assessed by an orthopaedic surgeon, and 39.3% had undergone surgery (i.e., arthroscopy or ACL reconstruction).

**Table 2:** Participant characteristics and reference standard availability (*n* = 725)

Variable	Summary Statistic
Participant Characteristic	
Sex: total (%) female	346 (47.7)
Age: years	26 (15–45)
Height: cm	173 (151–199)
Weight: kg	77.6 (43.0–152.4)
BMI: kg/m^2^	25.5 (17.5–51.0)
Not sport-related injury: total (%)	97 (13.4)
Sport: total (%) soccer	176 (24.3)
Time from injury to consultation: days	176 (1–798)
Reference Standard	
MRI°: total (%)	651 (89.8)
Orthopaedic surgeon exam: total (%)	494 (68.1)
Surgery: total (%)	285 (39.3)

Values represent median (minimum-maximum) unless otherwise stated. °266 participants ACL status was determined by MRI only

*BMI* Body Mass Index, *cm* centimeter, *kg* kilograms, *m* meter, *MRI* Magnetic Resonance Imaging

Of the 725 EMRs that met the inclusion criteria for the study, 436 (60.1%) represented a patient with a full-thickness ACL tear (ACL^+^) based upon the reference standard. A summary of demographic characteristics, potential diagnostic variables, and treatment pathway information by ACL status is presented in Table 3. Sex, age and body mass index did not differ between study groups. Although no single diagnostic indicator emerged, on average a greater proportion (*p* < 0.001) of the ACL^+^ study group reported a non-contact or plant-pivot mechanism of injury; a ‘popping sensation’, pain, immediate swelling, instability or inability to continue their activity at the time of injury; instability and an inability to return to activity at some point since the injury, and a family history of ACL tear compared to the ACL^−^ group. On clinical exam, a greater portion of those in the ACL^+^ group demonstrated a positive Lachman, positive pivot shift, or positive posterior drawer test compared to the ACL^−^ group.

**Table 3:** Demographic characteristics, potential diagnostic variables and treatment pathway by ACL status (*n* = 725)

Variable	ACL^−^ *n* = 289	ACL^+^ *n* = 436	*P*-value	Records Available
Participant Characteristic				
Sex: total (%) female	133 (46.0)	213 (48.9)	0.455	725
Age: median years (min-max)	26 (15–46)	26 (15–45)	0.989	725
Height: median cm (min-max)	176.4 (155–197)	172.0 (151–199)	< 0.001*	510
Weight: median kg (min-max)	81.0 (48.4–152.4)	76 (43.0–149.3)	0.003	510
BMI: median kg/m^2^ (min-max)	25.8 (18.4–45.4)	25.4 (17.5–51.0)	0.193	510
Not sport-related: total (%)	64 (22.1)	33 (7.6)	< 0.001*	725
Sport: total (%) soccer	47 (16.3)	129 (29.6)	< 0.001*	725
Mechanism of Injury				
Non-contact: % (95%CI)	72.4 (66.0,78.0)	84.2 (80.4,87.4)	< 0.001*	632
Hyperextension: % (95%CI)	10.8 (6.7,17.0)	11.8 (8.5,16.2)	0.759	420
Hyperflexion: % (95%CI)	6.1 (3.2,11.3)	1.8 (0.8,4.4)	0.021	420
Plant/pivot: % (95%CI)	17.7 (12.4,24.7)	52.0 (46.1,57.8)	< 0.001*	431
Valgus: % (95%CI)	23.2 (17.1,30.6)	27.7 (22.7,33.3)	0.313	422
Varus: % (95%CI)	5.3 (2.7,10.3)	7.3 (4.7,11.1)	0.436	424
Time of Injury				
Pop: % (95%CI)	47.3 (40.5,54.2)	83.3 (79.1,86.9)	< 0.001*	559
Pain: % (95%CI)	65.3 (58.1,72.0)	97.8 (95.3,99.0)	< 0.001*	461
Immediate swelling: % (95%CI)	57.1 (50.8,63.2)	91.8 (88.7,94.1)	< 0.001*	648
Instability: % (95%CI)	55.4 (47.2,63.3	97.5 (95.1,98.7)	< 0.001*	468
Locking: % (95%CI)	21.3 (13.5,31.8)	57.9 (34.8,78.0)	0.003	99
Catching: % (95%CI)	1.6 (0.2,10.7)	12.5 (1.4,58.2)	0.211	72
Inability to continue activity: % (95%CI)	51.8 (44.2,59.2)	93.2 (89.7,95.7)	< 0.001*	452
Clinical History (since the injury)				
Delayed pain: % (95%CI)	3.4 (1.5,7.4)	1.4 (0.5,3.8)	0.147	454
Delayed swelling: % (95%CI)	17.4 (13.1,22.8)	6.5 (4.5,9.4)	< 0.001*	641
Pain: % (95%CI)	100	100	–	668
Swelling: % (95%CI)	96.7 (90.2,98.9)	99.5 (97.8,99.9)	0.056*	457
Instability: % (95%CI)	75.4 (69.1,80.6)	98.8 (97.2,99.5)	< 0.001*	648
Locking: % (95%CI)	32.0 (25.2,39.8)	45.6 (37.4,54.1)	0.017	295
Catching: % (95%CI)	38.9 (28.2,50.7)	33.3 (21.6,47.5)	0.529	123
Inability to weight bear: % (95%CI)	93.0 (82.5,97.4)	99.4 (95.8,99.9)	0.016	222
Inability to return to activity: % (95%CI)	90.6 (85.3,94.0)	100	< 0.001*	546
Family history of ACL tear: % (95%CI)	3.3 (1.6,6.4)	22.8 (18.8,27.4)	< 0.001*	609
Clinical Examination				
Positive Lachman: % (95%CI)	9.6 (6.6,13.8)	91.3 (88.2,93.6)	< 0.001*	695
Positive Pivot shift: % (95%CI)	3.7 (1.8,7.1)	87.2 (83.3,90.3)	< 0.001*	570
Positive Anterior drawer: % (95%CI)	6.1 (3.6,10.0)	86.6 (81.2,90.7)	< 0.001*	433
Positive Posterior drawer: % (95%CI)	4.9 (2.8,8.4)	1.2 (0.5,0.3)	< 0.001*	649
Positive Valgus stress: % (95%CI)	9.7 (6.7,13.8)	7.4 (5.2,10.3)	0.284	690
Positive Varus stress: % (95%CI)	1.2 (0.4,3.6)	0.1 (0.04,2.6)	0.542	664
Care Pathway				
X-ray: total (%)	240 (83.0)	372 (85.3)	0.41	725
MRI: total (%)	279 (96.5)	356 (81.7)	< 0.001*	725
Orthopaedic surgeon exam: total (%)	103 (35.6)	391 (89.7)	< 0.001*	725
Surgery: total (%)	27 (9.3)	258 (59.2)	< 0.001*	725
Injury to consultation: median days (min-max)	126 (1–730)	176 (1–798)	0.0246	725
Injury to MRI: median days (min-max)	113 (2–804)	52 (1–535)	< 0.001*	725
Injury to surgeon: median days (min-max)	186 (1–806)	198 (10–806)	0.561	725
Injury to surgery: median days (min-max)	192 (15–988)	308 (19–959)	0.04	725

Values represent number (%) unless otherwise stated

*ACL* Anterior Cruciate Ligament, *ACL*^*−*^ ACL intact, *ACL*^*+*^ ACL full-thickness tear, *AKIC* Acute knee injury clinic, *BMI* Body Mass Index, *CI* confidence interval, *cm* centimeters, *kg* kilograms, *m* meters, *MRI* Magnetic Resonance Imaging

*Statistically significant at α < 0.001

The healthcare practitioner survey was completed by 17 clinicians (8 physiotherapists, 6 primary care sport and exercise medicine physicians, and 3 orthopaedic surgeons) with a median (minimum-maximum) of 11 (4–43) years of clinical experience. Across respondents, the most commonly selected potential diagnostic criteria for an ACL tear included: 1) positive Lachman test (65% of respondents ranked as the most important clinician-generated diagnostic criteria), 2) hearing or feeling a ‘pop’ at the time of injury (59% of respondents ranked as most important time of injury diagnostic criteria), 3) patient-reported knee instability since the injury (59% ranked as the most important clinical history diagnostic criteria), 4) plant/pivot mechanism of injury (53% ranked as the most important mechanism of injury diagnostic criteria) and, 5) age less than 25 years (47% ranked as the most important participant characteristic diagnostic criteria).

Table 4 summarizes the variables prioritized for univariable logistic regression by descending rank order based on the cumulative number of EMRs with available data by study group (ACL^+^ and ACL^−^). In keeping with our sample size calculation, univariable logistic regression was performed on 497 (68.6%) EMRs (321 ACL^+^ and 176 ACL^−^) containing complete data for both prioritized (age, sex, sport-related injury, Lachman test result, posterior drawer test result, family history of ACL tear and immediate swelling at the time of injury) and incidental (i.e., variables available in the data that did not meet selection criteria) variables (time between injury and assessment, valgus stress test result, varus test result). Despite being significant on univariable analyses, or ranking high on healthcare practitioner survey, feeling or hearing a ‘pop’ at the time of injury, mechanism of injury, patient-reported knee instability since the injury, or anterior drawer and pivot shift test result were inconsistently reported across EMRs and were not included due to a lack of data.

**Table 4:** Variables prioritized for univariable logistic regression based on univariable statistics, practitioner survey, literature review and data availability by study group

Prioritized Variable		Incidental Variable^+^	Total EMRs	ACL^+^ EMRs	ACL^−^ EMRs
Included	Age		725	444	281
	Sex		725	444	281
	Sport-related injury		725	444	281
		Time between injury and consultation	725	444	281
		Valgus stress test result	690	427	263
	Lachman test result		672	418	254
		Varus stress test result	647	406	241
	Posterior drawer test result		627	397	230
	Family history of ACL tear		550	343	207
	Immediate swelling		497	321	176
Excluded	History of knee instability		452	312	140
	Pivot shift test result		400	270	130
	Contact vs. non-contact		352	263	89
	Pop at time of injury		305	230	75
	History of inability to RTS		261	206	55

^+^Incidental variable = a variable available in the dataset despite not being prioritized based on univariate between group comparison, practitioner survey or literature review

*ACL* Anterior Cruciate Ligament, *ACL*^*+*^ ACL full-thickness tear, *ACL*^*−*^ ACL intact, *EMRs* electronic medical records, *RTS* return to sport

The results of univariable logistic regression models assessing the relationship between potential individual predictors and ACL status are presented in Table 5. Variables that were significantly associated with ACL tear at *p* < 0.1 included age (*p* = 0.001), sport-related injury or trauma (*p* = 0.094), family history of ACL tear (*p* = 0.032), immediate swelling at time of injury (*p* = 0.001) and Lachman test result (*p* < 0.001).

**Table 5:** Univariable logistic regression models (*n* = 497, ACL^+^ = 316, ACL^−^ = 181) assessing in the odds (95%CI) of an ACL tear

Variable	Reference	Estimate (SE)	OR (95% CI)	*P*-value
Age (years)	–	0.08 (0.03)	1.08 (1.03,1.14)	0.001
Sex (male/female)	Male	−0.21 (0.21)	0.66 (0.29,1.47)	0.306
Sport-related injury (sport/ non-sport)	Sport	0.54 (0.32)	2.97 (0.83,10.60)	0.094
Time between injury and consultation (days)	–	−0.0005 (0.001)	1.00 (0.99,1.00)	0.762
Valgus stress test (+/−)	+	−0.19 (0.46)	0.68 (0.11,4.20)	0.679
Lachman test result (+/−)	+	2.60 (0.22)	181.99 (75.73,437.31)	< 0.001
Varus stress test (+/−)	+	6.09 (586.8)	> 999.99 (< 0.001,> 999.99)	0.992
Posterior drawer test (+/−)	+	0.14 (0.54)	1.32 (0.16,11.06)	0.796
Family history (yes/no)	Yes	0.76 (0.35)	4.55 (1.14,18.15)	0.032
Immediate swelling (yes/no)	Yes	0.81 (0.25)	5.02 (1.92,13.14)	0.001

*ACL* Anterior Cruciate Ligament, *ACL*^*+*^ ACL full-thickness tear, *ACL*^*−*^ ACL intact, *SE* standard error, *OR* odds ratio, *CI* confidence interval, + = positive test result, − = negative test result

The results of the multivariable logistic regression models are summarized in Table 6. The first model included patient-reported variables (age, sport-related injury, immediate swelling, and family history ACL tear) only, while the second model included a combination of patient-reported (age, sport-related injury, immediate swelling, and family history ACL tear) and clinician-generated variables (Lachman test result). In the patient-reported model older age, sport-related injury, immediate swelling, and family history of ACL tear was found to be significantly associated with a full-thickness ACL tear diagnosis. When the Lachman test result was considered alongside the patient-reported variables in the combined model, only older age, immediate swelling, and a positive Lachman test result were significantly associated with the diagnosis of a full-thickness ACL tear.

**Table 6:** Multivariable logistic regression models (based on training data)

Variable	Condition	Model 1 (Patient-reported only)			Model 2 (Patient-reported and Clinician-generated)		
		Estimate (SE)	OR (95% CI)	*p*-value	Estimate (SE)	OR (95% CI)	*p*-value
Age	Year	0.047 (0.018)	1.05 (1.01.1.09)	0.007*	0.059 (0.028)	1.06 (1.00,1.12)	0.034*
Sport-related	Sport	1.261 (0.414)	3.53 (1.57,7.93)	0.002*	0.081 (0.701)	2.24 (0.57,8.85)	0.250
	Non-sport	Reference			Reference		
Family history	Yes	2.601 (0.616)	13.47 (4.03,45.06)	< 0.001*	1.021 (0.847)	2.78 (0.53,14.62)	0.228
	No	Reference			Reference		
Immediate swelling	Yes	2.532 (0.351)	12.58 (6.32,25.04)	< 0.001*	1.212 (0.543)	3.36 (1.16,9.74)	0.026*
	No	Reference			Reference		
Lachman test result	Positive	–	–	–	5.038 (0.498)	154.17 (58.04,409.49)	< 0.001*
	Negative	–	–	–	Reference		

*ACL* Anterior Cruciate Ligament, *SE* standard error, *OR* odds ratio, *CI* confidence interval

*p < 0.05

Model performance based on a test set (random 30% of data) is summarized in Table 7. It is interesting to note that the performance of the Lachman test alone was comparable to the combined patient-reported and clinician-generated variable model [accuracy rate 94.0% (95%CI 88.9,97.2), AUC (0.94), sensitivity 0.94 (95%CI 0.91,0.99), specificity (0.94 (95%CI 0.76,0.96), positive predictive value (0.94 (95%CI 0.88,0.98), negative predictive value (0.94 (95%CI 0.82,0.99), LR+ of 8.1 (95% CI 3.8,17.1), LR- of 0.03 (95%CI 0.01,0.10)].

**Table 7:** Multivariable logistic regression model performance (based on test set)

Performance Measure	Model 1 (Patient-reported only)	Model 2 (Patient-reported and Clinician-generated)
Accuracy rate (%; 95% CI)	84.0 (77.1,89.5)	94.7 (89.8,97.7)
AUC score	0.86	0.97
Sensitivity (95%CI)	0.59 (0.44,0.74)	0.94 (0.88,0.98)
Specificity (95%CI)	0.95 (0.89,0.98)	0.95 (0.82,0.99)
Positive Predictive Value (95%CI)	0.95 (0.89,0.98)	0.95 (0.82,0.99)
Negative Predictive Value (95%CI)	0.60 (0.44,0.74)	0.94 (0.82,0.99)
Positive Likelihood Ratio (95%CI)	5.53 (2.46,12.44)	9.51 (4.14,21.84)
Negative Likelihood Ratio (95%CI)	0.19 (0.12,0.30)	0.03 (0.01,0.10)

*CI* confidence interval, *AUC* area under the curve

## Discussion

To the best of our knowledge this is the first study to assess the accuracy of combinations of patient-reported and clinician-generated variables to identify a first-time ACL tear that considers differences in practice patterns and competencies of primary point-of-care practitioners**.** Our findings demonstrate that a combination of patient-reported and clinician-generated variables, or Lachman test alone, are superior for detecting a full-thickness ACL tear compared to patient-reported variables alone. Specifically, increasing age, immediate swelling and Lachman test result accurately identified 95% of first-time ACL tears, the Lachman test along accurately identified 94% of first-time ACL tears, while a combination of increasing age, sport-related trauma, immediate swelling, and family history of an ACL tear accurately identified 84% of tears. These accuracy rates are higher than those previously reported [35] and have direct implications for clinical decision support tool development for use in primary point-of-care clinical practice settings.

### Building from previous investigations

By leveraging EMR data over a two-year period we were able to include a large sample of patients with a wide variation of knee conditions, age and time since injury. Similar to previous investigators [33–35, 43], we have demonstrated that a combination of clinical history and examination variables is diagnostically superior to individual elements [24, 35, 39], and that immediate swelling [24, 35, 39] and the Lachman test result [33, 35] are important diagnostic criteria for an ACL tear. Further, we have confirmed the importance of trauma (in particular sport-related) for the diagnosis of an ACL tear previously reported by Decary et al. [35], and that when considered individually, a Lachman test may be useful (LR+ between 5 and 10) for diagnosing, and almost conclusive (LR- between < 0.1) for excluding an ACL tear when performed by clinicians with advanced orthopaedic training [44].

In contrast to past studies [45, 46], we have identified that increasing age and a family history of an ACL tear, may be important diagnostic criteria for an ACL tear. Interestingly, other variables previously reported to be useful for diagnosis including pivoting mechanism of injury, ‘popping’ sensation at the time of injury, giving away and anterior drawer or pivot shift test result [24, 35, 39, 43] did not factor into our findings. Reasons for this may include: previous studies smaller sample sizes [24, 34, 39]; differences in sample characteristics (participant age, acuteness and diversity of knee conditions); number of practitioners; practitioner characteristics (profession, experience, degree of confidence in performing and interpreting clinical tests) and; inability to assess specific variables (age, family history, ‘popping’ sensation or pivoting mechanism of injury) due to insufficient or lack of data availability.

### Implications for primary care

Primary point-of-care practitioners diagnosis ACL tears differently than practitioners with specialized orthopaedic training, relying more heavily on clinical history than a physical examination [24]. This likely reflects the unique challenges that primary care practitioners face in assessing acute knee injuries including an increased likelihood that pain, swelling and muscle guarding will interfere with the physical examination given the close proximity of the examination to the trauma. Varying levels of practitioner confidence in performing special clinical tests such as the Lachman or Pivot Shift test due to less orthopaedic training [47–49], and less exposure to patients with traumatic knee injuries may also be a challenge unique to primary point-of-care practitioners. Embracing these realities is essential to improving the diagnostic accuracy of ACL tears in primary care settings. Although our findings indicate that a combination of patient-reported variables (age, sport-related trauma, immediate swelling, and family history ACL tear) had inferior diagnostic accuracy than when considered alongside the Lachman test, they were capable of identifying 95% (specificity) of those without an ACL tear. Implementation of this information through the development of a primary point-of-care clinical decision support tool presents a unique opportunity to improve the efficiency of ACL tear diagnosis in primary care settings, while triaging patients to the most appropriate diagnostic (i.e., clinician with advanced orthopaedic training) and/or care pathway (i.e., rehabilitation and/or surgical consultation).

### Strengths and limitations

By leveraging the EMRs of a large, regional sports medicine clinic and multiple clinicians, we were able to investigate diagnostic indicators of ACL tear in the largest sample size reported to date. Despite the large number of records with ACL tears, it is important to acknowledge that this was a retrospective chart review and the data were not collected for the stated purpose. As a result, there were a large number of incomplete EMRs which made it difficult to include variables that others have identified as being helpful in diagnosing an ACL tear such as a ‘popping’ sensation at the time of injury, a pivot mechanism of injury and Pivot Shift Test result in our multivariable analyses [35]. It is also important to point out that these findings may not be generalizable to other practice settings, and older patients with non-sport related knee injuries. Despite these limitations, this unique real-world dataset provides an understanding of what variables may be most useful for diagnosing an ACL tear in a practice setting with a high prevalence of ACL tears and clinicians with orthopaedic training. This information lays the foundation for the development of clinical decision tools for primary point-of-care practitioners.

### Future steps

Beyond delivering two preliminary predictive statistical models, this research has allowed us to explore the quality and completeness of the EMR data, observe clinical processes and foster clinical relationships and collaborations. This information will directly inform a multi-site prospective study aimed at developing, externally validating and assessing the accuracy of a clinical decision support tool for identifying patients with full-thickness ACL tears in primary point-of-care settings. Once developed, this tool will provide a foundation for optimizing the care, development of appropriate clinical pathways, and outcomes of patients with acute knee injuries including an ACL tear.

## Conclusions

A combination of patient-reported and clinician-generated variables are superior for detecting a full-thickness ACL tear compared to patient-reported variables alone. Despite this, a high proportion of individuals without an ACL tear can be accurately identified by considering patient-reported age, injury setting, immediate swelling and family history of ACL tear. These findings directly inform future prospective development of a primary point-of-care clinical decision support tool to facilitate timely and accurate ACL tear diagnosis.

## Supplementary information

**Additional file 1.** Included International Classification of Disease 9 Codes.

**Additional file 2.** Practitioner Survey of Variables Important for ACL Tear Diagnosis.

## Data Availability

The datasets used and/or analysed during the current study are available from the corresponding author on reasonable request.

## Abbreviations

ACL: Anterior cruciate ligament
AKIC: Acute knee injury clinic
AUC: Area under the curve
BMI: Body mass index
CI: Confidence interval
cm: Centimeter
EMR: Electronic medical record
ICD: International Classification of Disease
kg: Kilograms
LR: Likelihood ratio
m: Meter
MRI: Magnetic resonance imaging
OA: Osteoarthritis
OR: Odds ratio
RTS: Return to sport
ROC: Receiver-operating characteristic
SE: Standard error
STARD: Standards for Reporting Diagnostic Accuracy Studies
