# Supplementary material for: Towards improving the identification of anterior cruciate ligament tears in primary point-of-care settings
Source: BMC Musculoskelet Disord. 2020 Apr 17;21:252. doi: 10.1186/s12891-020-03237-x (PMC7165371; doi:10.1186/s12891-020-03237-x)
Supplement: Supplementary file 1 — Additional file 1. Included International Classification of Disease 9 Codes. [file 12891_2020_3237_MOESM1_ESM.pdf]

ADDITIONAL FILE 1: Included International Classification of Disease 9 Codes

- All **717** codes (internal derangement of knee): **717.0-717.99**

- **719** (other and unspecified disorder of joint): **719.0** (effusion of joint), **719.4** (pain in joint), **719.47** (pain in joint in lower leg), **719.5** (stiffness of joint, not elsewhere classified), **719.56** (stiffness of joint in lower leg), **719.6** (other symptoms referable to joint), **719.66** (other symptoms referable to joint in lower leg), **719.8** (other specified disorders of joint), **719.86** (other specified disorders of joint in lower leg)

- **727** (other disorders of synovium, tendon and bursa): **727.0** (synovitis and tenosynovitis), **727.06** (synovitis and tenosynovitis in lower leg), **727.3** (other bursitis), **727.36** (other bursitis in lower leg), **727.5** (rupture of synovium), **727.56** (rupture of synovium in lower leg), **727.6** (rupture of tendon, non-traumatic), **727.66** (rupture of tendon in lower leg, non-traumatic), **727.8** (other disorders of synovium, tendon, and bursa), **727.86** (other disorders of synovium, tendon, and bursa in lower leg)

- **728** (disorders of muscle, ligament, fascia): **728.4, 72** (laxity of ligament) **728.46** (laxity of ligament in lower leg), **728.5** (hypermobility syndrome), **728.56** (hypermobility syndrome in lower leg), **728.8** (other disorders of muscle, ligament and fascia), **728.86** (other disorders of muscle ligament and fascia in lower leg)

- **729** (other disorders of soft tissues): **729.5** (pain in limb), **729.56** (pain in lower leg),**729.8** (other symptoms referable to limbs), **729.86** (other symptoms referable to lower leg), **729.9** (other and unspecified disorders of soft tissue), **729.96** (other and unspecified disorders of soft tissue in lower leg)

- **836** (dislocation of knee): **836.0** (tear of medial cartilage or meniscus of knee, current), **836.1** (tear of lateral cartilage or meniscus of knee, current), **836.2** (other tear of cartilage or meniscus of knee, current), **836.3** (dislocation of patella, simple), **836.4** (dislocation of patella, compound), **836.5** (other dislocation of knee, simple), **836.6** (other dislocation of knee, compound)

- **844** (sprain and strains of knee and leg): **844.0** (lateral collateral ligament of knee), **844.1** (medial collateral ligament of knee), **844.2** (sprain of cruciate ligament of knee), **844.3** (tibiofibular (joint) (ligament), superior), **844.8** (other specified sites of knee and leg), **844.9** (unspecified).
