# Supplementary material for: Towards improving the identification of anterior cruciate ligament tears in primary point-of-care settings
Source: BMC Musculoskelet Disord. 2020 Apr 17;21:252. doi: 10.1186/s12891-020-03237-x (PMC7165371; doi:10.1186/s12891-020-03237-x)
Supplement: Supplementary file 2 — Additional file 2. Practitioner Survey of Variables Important for ACL Tear Diagnosis. [file 12891_2020_3237_MOESM2_ESM.pdf]

Additional File #2: Practitioner Survey of Variables Important for ACL Tear Diagnosis

As we develop a clinical decision tool to assist in the diagnosis of ACL tears we would very much like to incorporate the expertise of the clinicians at the XXXXXXXXXXX.

To that end we have created an anonymous short survey in which you will CHOOSE and RANK items that you think are the most important for diagnosing an ACL tear. Keep in mind that the items included in the survey are based upon the initial informal feedback that some of you had provided at the very start of this project.

For all sections please RANK the items that you feel are the most important for diagnosing an ACL tear. If you do not think that an item is important for diagnosing an ACL tear you do not have to rank it.

Please choose the response that most closely describes your role in patient care	◯ Sport and Exercise Medicine Physician<br>◯ Orthopaedic Surgeon<br>◯ Physiotherapist<br>◯ I prefer not to say
How many years have you been practicing in your current profession?	◯ 0-5 years<br>◯ 6-10 years<br>◯ 11-15 years<br>◯ 15-20 years<br>◯ >21 years<br>◯ I prefer not to say
What is your sex?	◯ Female<br>◯ Male<br>◯ I prefer not to say
What is your age?	________________<br>(i.e., 35)

Please rank the following PATIENT CHARACTERISTICS based on what you feel to be the most important for diagnosing an ACL tear. If you do not think that an item is important for diagnosing an ACL tear you do not have to rank it.

NOTE: You will only be able to pick one patient characteristic for each ranking (i.e., 1-4)

		1	2	3	4
1	Injury age < 25 years	○	○	○	○
2	Female sex	○	○	○	○
3	Body Mass Index >30 (Obese)	○	○	○	○
4	Time between injury and clinical assessment	○	○	○	○

Please list any other patient characteristics that you feel are important for diagnosing an ACL tear.

____

Please rank (1 through 7) the following MECHANISM OF INJURY CHARACTERISTICS based on what you feel to be the most important for diagnosing an ACL tear. If you do not think that an item is important for diagnosing an ACL tear you do not have to rank it.

NOTE: You will only be able to pick one mechanism of injury for each ranking (i.e., 1-7)

		1	2	3	4	5	6	7
1	Injury occurred during sport participation	◯	◯	◯	◯	◯	◯	◯
2	Non-contact mechanism of injury	◯	◯	◯	◯	◯	◯	◯
3	Hyperextension mechanism of injury	◯	◯	◯	◯	◯	◯	◯
4	Hyperflexion mechanism of injury	◯	◯	◯	◯	◯	◯	◯
5	Plant pivot mechanism of injury	◯	◯	◯	◯	◯	◯	◯
6	Valgus mechanism of injury	◯	◯	◯	◯	◯	◯	◯
7	Varus mechanism of injury	◯	◯	◯	◯	◯	◯	◯

Please list any other mechanism of injury characteristics that you feel are important for diagnosing an ACL tear. ________________________________

Please rank (1 through 9) the following PATIENT REPORTED SYMPTOMS from THE TIME OF INJURY based on what you feel to be the most important for diagnosing an ACL tear. If you do not think that an item is important for diagnosing an ACL tear you do not have to rank it.

NOTE: You will only be able to pick one patient reported symptom from the time of injury for each ranking (i.e., 1-9)

		1	2	3	4	5	6	7	8	9
1	Patient heard a 'pop'	○	○	○	○	○	○	○	○	○
2	Patient reported immediate pain (< 30 min)	○	○	○	○	○	○	○	○	○
3	Patient reported delayed pain (>30 min)	○	○	○	○	○	○	○	○	○
4	Patient reported immediate swelling (hemarthrosis < 4 hrs)	○	○	○	○	○	○	○	○	○
5	Patient reported delayed swelling (hemarthrosis >4 hrs)	○	○	○	○	○	○	○	○	○
6	Patient reported knee locking	○	○	○	○	○	○	○	○	○
7	Patient reported knee instability	○	○	○	○	○	○	○	○	○
8	Patient reported knee catching	○	○	○	○	○	○	○	○	○
9	Patient reported they could not return to sport	○	○	○	○	○	○	○	○	○

Please list any other patient symptoms reported at the time of injury that you feel are important for diagnosing an ACL tear. __________________________________

Please rank (1 through 10) the following CLINICAL HISTORY RESPONSES based on what you feel to be the most important for diagnosing an ACL tear. If you do not think that an item is important for diagnosing an ACL tear you do not have to rank it.

NOTE: You will only be able to pick one clinical history response for each ranking (i.e., 1-10)

		1	2	3	4	5	6	7	8	9	10
1	History of knee instability	○	○	○	○	○	○	○	○	○	○
2	History of knee pain	○	○	○	○	○	○	○	○	○	○
3	History of knee catching	○	○	○	○	○	○	○	○	○	○
4	History of knee locking	○	○	○	○	○	○	○	○	○	○
5	History of being unable to fully weight-bear for 3 steps	○	○	○	○	○	○	○	○	○	○
6	History of knee joint effusion	○	○	○	○	○	○	○	○	○	○
7	History of being unable to return to sport due to their knee injury	○	○	○	○	○	○	○	○	○	○
8	History of reduced knee flexion range of motion	○	○	○	○	○	○	○	○	○	○
9	History of reduced knee extension range of motion	○	○	○	○	○	○	○	○	○	○
10	Family history of ACL tears	○	○	○	○	○	○	○	○	○	○

Please list any other clinical history responses that you feel are important for diagnosing an ACL tear.

____

Please rank (1 through 9) the following CLINICAL EXAM TESTS based on what you feel to be the most important for diagnosing an ACL tear. If you do not think that an item is important for diagnosing an ACL tear you do not have to rank it.

NOTE: You will only be able to pick one clinical exam test for each ranking (i.e., 1-9)

		1	2	3	4	5	6	7	8	9
1	Lachman test	○	○	○	○	○	○	○	○	○
2	Pivot shift test	○	○	○	○	○	○	○	○	○
3	Anterior drawer test	○	○	○	○	○	○	○	○	○
4	Posterior drawer test	○	○	○	○	○	○	○	○	○
5	Vagus (MCL) stress test	○	○	○	○	○	○	○	○	○
6	Varus (LCL) stress test	○	○	○	○	○	○	○	○	○
7	McMurray test	○	○	○	○	○	○	○	○	○
8	Apprehension test	○	○	○	○	○	○	○	○	○
9	Tenderness on palpation - joint line	○	○	○	○	○	○	○	○	○

Please list any other clinical exam tests that you feel are important for diagnosing an ACL tear.

____

Are there any other things that you feel are important to consider or that are common to the clinical presentation associated with ACL tears that you would like to add? __________________________________
